# A Systematic Review on Applications of Artificial Intelligence for Obesity Prevention

**DOI:** 10.1111/obr.70062

**Published:** 2025-12-23

**Authors:** Atefehsadat Haghighathoseini, Shuo‐yu Lin, Ge Song, Ruopeng An, Hong Xue

**Affiliations:** ^1^ Department of Health Administration and Policy, College of Public Health George Mason University Fairfax Virginia USA; ^2^ Constance and Martin Silver Center on Data Science and Social Equity, Silver School of Social Work New York University New York City New York USA

**Keywords:** artificial intelligence (AI), obesity, prevention, systematic review

## Abstract

This systematic review examines the applications of artificial intelligence (AI) in preventing obesity, addressing a critical public health issue that affects a substantial portion of the population. With obesity rates rising alarmingly, particularly in the United States, this review synthesizes findings from 46 studies published between 2008 and 2024, highlighting the potential of AI technologies to enhance obesity prevention efforts. The review employs PRISMA guidelines to ensure a rigorous methodology, encompassing a comprehensive search of major biomedical databases. The results indicate a notable increase in research activity since 2018, with a predominant focus on AI‐driven methodologies for obesity detection, whereas areas such as prevention, management, and treatment remain underexplored. Various machine learning (ML) and deep learning (DL) algorithms, including support vector machines and long short‐term memory networks, were identified, with performance metrics such as accuracy and sensitivity commonly reported. Despite the promising advancements, the review identifies significant gaps in the literature, including a lack of comprehensive frameworks for integrating AI in real‐world settings and the need for more targeted research on prevention strategies. This review underscores the transformative potential of AI in combating obesity and calls for further investigation to optimize its applications in public health initiatives.

## 1 Introduction

Obesity is an epidemic with influential consequences for public health, economics, and society. In the United States, obesity affects over 42% of adults, causing an estimated annual healthcare expenditure exceeding $190 billion [1, 2]. This burden is compounded by social determinants, including socioeconomic disparities, limited access to healthcare, and community environments that foster unhealthy lifestyles [3]. Addressing obesity requires a multifaceted approach, emphasizing prevention, management, and treatment strategies that are equitable, effective, and sustainable [4].

The advent of artificial intelligence (AI) offers a transformative opportunity to tackle the obesity epidemic. By leveraging advanced algorithms and computational power, AI can process vast datasets to uncover risk factors, predict trends, and design personalized interventions [5]. For example, machine learning (ML) and deep learning (DL) algorithms have shown promise in identifying obesity‐related patterns with high precision, enabling early intervention. Furthermore, AI‐powered tools such as wearable devices and mobile health applications enhance real‐time monitoring and feedback, empowering individuals to make healthier choices [6, 7]. As AI continues to evolve, its integration into healthcare systems presents a unique opportunity to revolutionize the management and prevention of obesity.

Despite the promising potential of AI in combating obesity, a significant gap remains in the literature regarding its application in this domain. Most studies have focused on obesity detection, leaving prevention and management underexplored [8]. Integrating AI into real‐world healthcare settings faces challenges, including data standardization, model generalizability, and ethical considerations [9]. Additionally, there is a lack of comprehensive frameworks that outline the methodologies and best practices for integrating AI into obesity prevention strategies [10].

This systematic review aims to synthesize existing literature on AI applications in obesity prevention, highlight successful methodologies, and propose future directions for research and practice. By addressing these gaps, we aim to enhance the role of AI in combating the obesity epidemic and strengthening public health outcomes.

## 2 Methodology

This systematic review was conducted in accordance with the PRISMA (Preferred Reporting Items for Systematic Reviews and Meta‐analysis) guidelines to ensure transparency and reproducibility. The review covered both medical and technical aspects of this topic. Two authors independently searched major biomedical research databases, including PubMed, Google Scholar, IEEE Xplore, and Web of Science, to identify studies on the applications of AI in obesity research. The reviewers initially screened all abstracts and assessed eligibility according to the inclusion and exclusion criteria. Complete manuscripts of eligible articles were then reviewed to select the final papers for inclusion. The search was performed within the titles, abstracts, and keywords of the manuscripts using terms such as “artificial intelligence,” “machine learning,” “deep learning,” “neural networks,” “obesity prevention,” “overweight prevention,” and “body mass index control.” Boolean operators (AND, OR) were used to combine these terms effectively. Truncation and wildcards facilitated the search for word variations, such as using “obes*” to encompass “obese,” “obesity,” and related terms. Additionally, database‐specific controlled vocabulary, like MeSH terms “Artificial Intelligence” and “Obesity Prevention” in PubMed, was employed.

The inclusion criteria for article selection were as follows: (a) articles addressed AI applications in obesity research; (b) articles described the use of AI techniques such as machine learning, deep learning, or neural networks; (c) articles presented quantitative outcomes related to obesity prevention; and (d) articles were written in English. Articles presented the following characteristics were excluded from the review: (a) Articles did not involve any form of AI or machine learning; (b) articles did not focus on obesity or weight management; (c) articles concentrated exclusively on childhood obesity, as our primary interest lay in AI applications for adult obesity; (d) articles did not report outcomes relevant to obesity or focus solely on treatment rather than broader management aspects; (e) articles focused on other health conditions, qualitative studies, reviews, case reports, and those without detailed AI methodology; and (f) nonempirical studies, including reviews, editorials, and opinion pieces unless they provided significant theoretical or methodological contributions to the field. These inclusion and exclusion criteria ensure that the systematic review focuses on the intersection of AI and obesity, offering a comprehensive overview of the current state of research in this area.

## 3 Results

**FIGURE 1:**
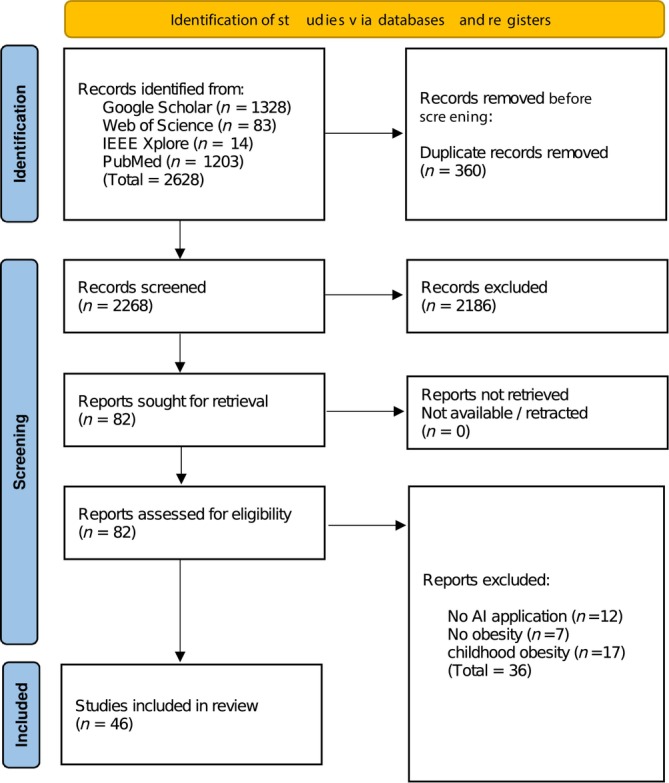
PRISMA flow diagram.

The PRISMA flow diagram shown in Figure 1 illustrates the article selection process, with 2628 articles initially identified from several databases and registers, including Google Scholar, Web of Science, IEEE Xplore, and PubMed. Before the screening process, 360 duplicate articles were removed, leaving 2268 to be screened. During the screening phase, a substantial number of articles, specifically 2186, were excluded. Following the screening, 82 reports were sought for retrieval, and all were successfully retrieved as none were unavailable or retracted. These reports were then assessed for eligibility, resulting in the exclusion of 36 reports due to various reasons: 12 reports had no AI application, seven reports did not focus on obesity, and 17 reports were related to childhood obesity. Finally, 46 studies were included in the review, providing a focused analysis of the relevant research. This rigorous process ensured that the studies included were pertinent and met the established criteria for the review.

Table 1 presents a detailed summary of study characteristics, study year, study design, sample size, study population, data source, outcome variable, independent variables, algorithms used, model performance metrics, and limitations. The studies were published over various years (2008–2024), with a predominant focus on machine learning (ML) and deep learning (DL) applications in obesity research, employing diverse study designs, including systematic reviews and participatory modeling approaches. Sample sizes varied widely, ranging from small cohorts of 19 participants to large datasets exceeding 600,000 records, indicating a broad range of research scales. The study populations primarily consisted of adults from diverse geographic regions, including Latin America and the United States, with data sourced from repositories such as Kaggle and the UCI. Most articles focused on obesity‐related outcomes, such as BMI and weight change. Independent variables were identified to encompass a wide range of factors, including demographic data, lifestyle habits, and psychological variables. At the same time, algorithms used included traditional ML methods like support vector machines (SVMs) and advanced DL techniques like long short‐term memory (LSTM) networks. Model performance metrics were typically evaluated using accuracy, sensitivity, and specificity, although limitations such as data bias and the representativeness of samples were commonly noted across studies.

**TABLE 1:** Summary of the characteristics of the included studies.

First author, year [reference]	Study design	Sample size	Study population	Data source	Outcome variable	Independent variables	Algorithms used	Model performance metrics	Limitations
Anar Taghiyev, 2020 [17]	Two‐stage hybrid machine learning approach to identify the causes of obesity among females aged 18 and above in Turkey	500	Females aged 18 years and above who applied to ASFHC from March 15, 2019, to November 1, 2019	Two sources: 1. Electronic Health Records (EHR) of Aksaray Sultanhani Family Health Center (ASFHC); 2. A questionnaire was administered to the participants.	Obesity, categorized based on body mass index (BMI) values: OBESE: BMI ≥ 30 kg/m^2^ and NONOBESE: BMI < 29.9 kg/m^2^	Sociodemographic characteristics (e.g., age, family type, marital status, education level). Health indicators (e.g., blood pressure, fasting blood glucose levels, and cholesterol levels). Lifestyle factors (e.g., physical activity, diet, smoking, and alcohol consumption). Anthropometric measurements (e.g., weight, height, waist circumference, and hip circumference).	Decision trees (DT) for feature selection in the first stage. Logistic regression (LR) for classification in the second stage	Evaluated using accuracy, sensitivity, specificity, precision, recall, and F measure	The study focused only on females aged 18 and above, which may not generalize to other age groups or genders. The data were collected from a single health center, limiting the generalizability of the findings to other regions or populations. The study relied on self‐reported data from questionnaires, which may introduce bias due to recall errors or social desirability. The model's performance was evaluated using a holdout validation method, which may not fully capture the model's predictive capabilities in different datasets.
K. W. DeGregory, 2018 [27]	Comprehensive review of machine learning methods applied to obesity‐related data, with a focus on demonstrating the application of these methods to the National Health and Nutrition Examination Survey (NHANES) dataset	25,367	US adults aged 18 years and older, with a focus on three race/ethnic groups: non‐Hispanic Whites, non‐Hispanic Blacks, and Mexican Americans	NHANES 1999–2006	High blood pressure High levels of percent body fat	Race Gender Waist circumference (cm) Education level Body mass index (BMI, kg/m^2^) Age (years)	Predictive models: Linear and logistic regression Neural networks Deep learning Decision tree analysis Descriptive models: Cluster analysis Principal component analysis Network science Topological data analysis	Area under the curve (AUC) receiver operating characteristic (ROC) curves	Predictive models: Regression: Assumptions about data distribution and functional form must be made, requiring knowledge of data properties and model capabilities. Neural networks and deep learning: Prone to overfitting with small sample sizes and require large datasets for optimal performance. Complex models may not be easily interpretable or accessible for immediate clinical application. Decision trees: Computational complexity and potential for suboptimal threshold selection due to the use of greedy algorithms. Descriptive models: Cluster analysis and principal component analysis: Not directly predictive but rather descriptive, requiring additional steps for hypothesis generation and prediction. Network science and topological data analysis: Require specific expertise and software, which may not be as widely accessible or standardized as more established methods. The structure of network layers must be specifically designated, potentially introducing bias.
Hui Yang, 2009 [11]	Hybrid text mining approach combining dictionary look‐up, rule‐based, and machine learning methods to predict disease statuses from clinical discharge summaries	507	Patients with obesity and associated comorbidities, focusing on 15 related diseases such as diabetes mellitus, hypertension, and coronary artery disease	Research Patient Data Repository of Partners Health Care and consisted of hospital discharge summaries	Disease status, indicating whether a patient was diagnosed with a specific disease (Y—yes, disease present), not diagnosed with the disease (N—no, disease absent), the diagnosis was questionable (Q—questionable), or the disease status was not mentioned in the discharge summary (U—unmentioned)	Explicit references to diseases in the narrative text and implicit evidence suggesting a disease status.	Textual prediction: Dictionary look‐up and rule‐based methods followed by contextual relabeling. Intuitive prediction: Combination of term and clinical inference rule matching for sentence‐level evidence extraction, and a support vector machine (SVM) classifier for document‐level classification	F measure and accuracy	Data limitations: The system's performance is dependent on the quality and representativeness of the training data. Manual analysis and statistical identification of clues in the training data are required, which can be labor‐intensive. Generalizability: Although the system's infrastructure is general enough to be reused across the clinical domain, some details require knowledge elicitation from domain experts or medical resources, and manual changes to the system may be necessary. Ambiguity handling: The system could be improved by expanding the set of clinical inference rules and enhancing its ability to dynamically expand abbreviations and handle ambiguous terms. Medication discriminative power: The estimation of the discriminative power of medications used to treat specific diseases could be improved to enhance intuitive predictions.
Magda RR Cruz, 2014 [31]	Computerized intelligent decision support system (DSS) using Bayesian networks	15	Patients who have undergone bariatric surgery, specifically the Roux‐en‐Y gastric bypass technique	Scientific literature, expert consultations, and case studies	Accuracy of the nutritional diagnosis provided by the DSS	Factors that influence nutritional diagnosis, such as gender, age, time since surgery, biochemical markers (e.g., hemoglobin, ferritin, and vitamin B12), food intake, and physical signs and symptoms of nutrient deficiency	The DSS employs Bayesian networks (BN), specifically implemented using Shell Netica.	Success rate, sensitivity, specificity, and ROC curve	Generalizability: The system's performance is validated for a specific group of patients (those who have undergone Roux‐en‐Y gastric bypass surgery). Its applicability to other types of bariatric surgery or patient populations may require further validation. Data source: The system relies on published literature and expert consultations for training. As new research emerges, the system may need to be updated to incorporate the latest findings. Subjectivity in data: Some of the data used for training and evaluation, such as dietary intake and physical signs/symptoms, are subjective and may vary based on individual professional judgment. Lack of comparison: There is no comparison with other DSSs in the field, as no similar systems are known to the authors.
Bo‐Yong Park, 2020 [50]	Longitudinal observational study using resting‐state functional magnetic resonance imaging (rs‐fMRI) data to predict changes in body mass index (BMI) in adolescents over a 1‐ to 2‐year period	76 Adolescents from the Enhanced Nathan Kline Institute Rockland Sample (NKI‐RS) database and an additional 22 adolescents and young adults from the Samsung Medical Center	Adolescents and young adults	Enhanced Nathan Kline Institute Rockland Sample (NKI‐RS) database and the Samsung Medical Center	Change in BMI (ΔBMI) between the first and second visits	Functional connectivity features derived from rs‐fMRI data, specifically the weighted degree centrality (DC) values of various brain regions	Least absolute shrinkage and selection operator (LASSO) framework for feature selection and a linear regression model for BMI prediction	Intraclass correlation (ICC) and the area under the receiver operating characteristic curve (AUC)	Sample size and diversity: The study had a relatively small sample size, and the subjects were predominantly of African American and White ethnicity. This limits the generalizability of the findings across different ethnic groups. Short follow‐up period: The time interval between the two visits was relatively short (1–2 years), which may not capture long‐term changes in BMI and associated cardiovascular diseases. Limited clinical variables: The study considered only a few clinical variables related to eating disorders, anxiety, and depression. Additional factors such as genetics, behavior, and environment could provide a more comprehensive understanding of BMI changes. Age groups and image acquisition settings: The study used data from two distinct age groups (preadolescents and young adults) with different image acquisition settings, which could introduce variability in the results. Model replicability: Although the model was replicated in a local dataset, further validation across larger and more diverse samples is needed to confirm the findings.
Sylvain Iceta, 2021 [45]	Retrospective study to develop a fast screening tool for emotional eating detection in patients with obesity using artificial intelligence (machine learning)	176	Adult patients who aged 18 and 70 with obesity hospitalized in a tertiary care center with body mass index (BMI) ≥ 35 kg/m^2^ with at least two obesity‐related comorbidities (e.g., Type 2 diabetes, hypertension, dyslipidemia, sleep obstructive apnea) or BMI ≥ 45 kg/m^2^	Electronically registered clinical anonymized data from January 2017 to January 2018	Food addiction (FA) diagnosis based on the Yale Food Addiction Scale (YFAS): FA‐positive (FA+) and FA‐negative (FA−) groups	Three key items with a fast correlation‐based filter (FCBF) score > 0.1: “I eat to forget my problems.” “I eat more when I'm alone.” “I eat sweets or comfort foods.”	Logistic regression, artificial neural networks, naïve Bayes classification, decision tree, AdaBoost meta‐algorithm, CN2 rule inducer algorithm, SVM algorithm, *k*‐nearest neighbors' algorithm, and stochastic gradient algorithm	Area under the curve (AUC) score and F1 measure	Sample size: The relatively small sample size for a data mining study Data source: Use of a retrospective design and absence of other measures of eating behaviors Specificity: It is not possible to determine the specificity of the test for food addiction or other close constructs. Validation: The study requires a validation study of the FAST nomogram in a larger population to confirm its clinical utility and relevance.
Milla Kibble, 2020 [47]	Integrative machine learning approach, specifically group factor analysis (GFA) to analyze multiple datasets simultaneously	43	43 Young adult MZ twin pairs (aged 22–36 years) from the TwinFat study, a substudy of The Finnish Twin Cohort (FTC)	Clinical, cytokine, genomic, methylation, and dietary data from the TwinFat study	Obesity	Clinical variables (e.g., BMI, waist circumference, and fat percentage), cytokine levels, genomic data (SNPs related to obesity), methylation data (CpGs associated with obesity‐related traits), and dietary information	Group factor analysis (GFA)	GFA	The inclusion of self‐reported measures for physical activity and eating habits, which may introduce bias. The challenge of objectively measuring lifetime history of smoking and other environmental exposures The decision to limit the number of SNPs and CpGs, which may exclude relevant genetic and epigenetic information The potential for future improvements by incorporating additional data sources, such as metabolic profiles and stress‐related measures
Alexander A. Huang, 2024 [54]	Cross‐sectional cohort study to improve the prediction of obesity and investigate its relationships with various factors	6146	Adults aged 18 and over with body mass index (BMI) ≥ 30	NHANES 2017–2020	Obesity	Demographic information: age, gender, race/ethnicity, income_poverty_ratio. Health and laboratory data: HS C‐reactive protein, insulin, blood lead, blood cadmium, uric acid, creatinine, and various other clinical measurements. Lifestyle factors: Dietary habits, exercise routines, mental health, and physical examinations	XGBoost (extreme gradient boosting), artificial neural networks, gradient boost modeling, and random forest	Area under the receiver operator curve (AUROC), sensitivity, specificity, precision, recall, prevalence, detection rate, detection prevalence, and balanced accuracy	Cross‐sectional design: The study provides a snapshot at a single point in time, limiting the ability to establish causality or temporal sequences. Recall and response bias: Potential biases inherent in survey‐based data collection methods. Generalizability: Although the study aims to represent the national population, the findings may not generalize to all populations because of the specific characteristics of the NHANES dataset. Causality: The study identifies associations but cannot definitively establish causal relationships. Further research is needed to explore the causal nature of the identified associations.
Mahmut Dirik, 2023 [22]	The study follows a structured methodology that includes data collection, refinement, implementation of machine learning models, performance evaluation, and analysis of results that aims to develop a predictive model for identifying overweight or obese individuals based on physical characteristics and dietary habits.	2111	Individuals from Mexico, Peru, and Colombia	The data source is a dataset referred to as “NObesity,” which includes information on dietary habits and physical conditions.	Level of obesity, which is classified into different categories such as underweight, normal weight, overweight level I, overweight level II, obesity type I, obesity type II, and obesity type III	Demographic information Anthropometric measurements Data on dietary habits Physical exertion	Multilayer perceptron (MLP) Support vector machine (SVM) Fuzzy *k*‐nearest neighbors (FuzzyNN) Fuzzy unordered rule induction algorithm (FURIA) Rough sets (RS) Random tree (RT) Random forest (RF) naïve Bayes (NB) Logistic regression (LR) Decision table (DT)	Accuracy Precision Recall F1 score ROC curves AUC Kappa statistic Mean absolute error (MAE) Root mean square error (RMSE)	Dataset limitations: The study relies on a specific dataset, and the generalizability of the findings may be limited to the characteristics of this dataset. Model complexity: Some machine learning models, such as random forest, can be complex and may require significant computational resources for training and evaluation. Data quality and representation: The accuracy of the predictions depends on the quality and representativeness of the data. If the data contains biases or is not representative of the broader population, the model's predictions may be affected. Interpretability: Some machine learning models, especially deep learning models, may be considered “black‐box” models, making it difficult to interpret how they make predictions. Overfitting: There is a risk of overfitting, where a model performs well on the training data but poorly on unseen data. Appropriate cross‐validation techniques are required to mitigate this risk. External validation: The models need to be externally validated on different datasets to ensure their robustness and applicability in real‐world scenarios.
Ruopeng An, 2022 [7]	The included studies adopted various designs, with the majority being cross‐sectional (70%), followed by prospective (15%), retrospective (13%), and one study using a cotwin control design (2%).	Sample sizes varied widely, with some studies having as few as 20 participants and others as many as 5,265,265. The distribution of sample sizes across the studies was as follows: 15% (7/46) had a sample size between 20 and 82. 24% (11/46) had a sample size between 130 and 600. 41% (19/46) had a sample size between 1061 and 9524. 13% (6/46) had a sample size between 16,553 and 49,805. 4% (2/46) had a sample size between 244,053 and 618,898. 2% (1/46) had a sample size of 5,265,265.	50% (23/46) of the studies focused on adults. 30% (14/46) focused on children and adolescents. 2% (1/46) focused on people of all ages. 17% (8/46) did not report the age range of participants.	Data sources were diverse, including health surveys, electronic health records, MRI scans, social media data, and geographically aggregated datasets.	Included obesity‐related measures such as anthropometrics (body weight, BMI, BFP, WC, and WHR) and biomarkers	Independent variables varied across studies and included demographic factors, socioeconomic factors, healthcare factors, environmental factors, anthropometric data, and various clinical variables.	Machine learning (ML) models: logistic regression (LR), multilayer perceptron (MLP), support vector machine (SVM), naïve Bayes (NB), decision tree (DT), multiple regression (MR), multivariate adaptive regression splines (MARS), extreme learning machine (ELM), random forest (RF), BayesNet (BN), gradient boosting (GB), *k*‐nearest neighbor (*k*‐NN), least absolute shrinkage and selection operator (LASSO), partial least squares (PLS), linear model (LM), principal component analysis (PCA), positive and unlabelled (PU), group factor analysis (GFA), multiobjective evolutionary fuzzy classifier (MEFC) Deep learning (DL) models: convolutional neural network (CNN), long short‐term memory (LSTM)	Accuracy	The limitations of the studies were not explicitly listed in the provided context. Common limitations in AI research generally include data quality issues, selection bias, overfitting, and lack of generalizability. The diversity of study designs, data sources, and outcomes may also limit the comparability of findings across studies.
Xiaobei Zhou, 2022 [34]	Minireview that focuses on the application of machine learning (ML) models in predicting and preventing obesity. It involves a semiautomated data collection method incorporating data mining techniques to identify relevant studies.	Not applicable as this is a review article. The review includes 25 open‐source ML algorithms or models applied in obesity research.	Not applicable as this is a review article. However, the review discusses ML applications in various segments such as diet and nutrition, physical activity, geographic environment, genetics or genomics, and microbiome.	The review uses data from the PubMed and Google Scholar databases, focusing on studies published from inception until March 2022.	Prediction and prevention of obesity. The review discusses various ML models that are used to predict obesity rates, identify risk factors, and optimize weight loss results.	The independent variables include various factors that contribute to obesity, such as dietary patterns, physical activity, geographic environment, genetic or genomic data, and microbiome composition.	The review discusses a range of ML algorithms, including deep learning approaches, knowledge graph systems, statistical methods, and geographically weighted models. Some of the specific algorithms mentioned include PFoodReq, FlavorGraph, DeepFood, Market2Dish, LC‐N2G, NutriGenomeDB, MapMetadataEnrichment, GWmodel, schema, mHealthDroid, MobileCoach, PGS Catalog, Impute.me, DeepVariant, NeuralCVD, DeepCOMBI, DeepMicro, DeepMicrobes, SortMeRNA, q2‐feature‐classifier, Swarm, GEDFN, MDeep, TaxoNN, MetaPheno, and others.	The review does not provide specific performance metrics for each model. However, it emphasizes the importance of open‐source sharing and reproducibility of ML models in obesity research.	The review highlights several limitations, including the complexity of obesity etiologies, the lack of integrated models that span multiple fields or data types, the limited development of open‐source nutrigenetic models, and the absence of open‐source ML models in commercial mobile health applications. Additionally, the review points out that most ML models in obesity‐related research are isolated in a single field or factor, and there is a need for future research to develop comprehensive models that integrate multiple data types across platforms.
Harold Edward Bays, 2023 [36]	CPS that discusses the potential applications of AI in obesity management.	As this is not a study, there is no sample size mentioned.	The focus of the document is on individuals affected by obesity, but it does not specify a particular study population or demographic.	Electronic health records (EHR)	Specific outcome variables are not mentioned. It discusses the potential of AI to improve various aspects of obesity management, such as telemedicine, data analysis, and personalized medicine but does not specify a primary outcome variable for analysis.	The document does not detail specific independent variables. It broadly discusses the use of AI to analyze various types of data, including patient demographics, medical history, and real‐time monitoring data but does not specify independent variables in the context of a research study.	Although the document discusses the use of AI and machine learning, it does not specify particular algorithms used. It mentions the development of algorithms and statistical models that enable computers to learn from data input, but does not provide specific names or types of algorithms.	The document does not provide specific model performance metrics. It discusses the potential benefits and challenges of using AI in obesity management but does not present empirical results or performance metrics.	Data bias: If the initial data input is not representative of the population, it can lead to flawed outputs. Algorithmic bias: Disproportionate design of algorithms can lead to nonobjective responses. Confirmation bias: Programming AI to confirm preexisting beliefs can reinforce stereotypes. Selection bias: Nonrandom selection of data can skew results. Attribution bias: Failure to consider alternative causes can lead to erroneous assumptions. Sampling bias: Nonrepresentative samples can produce flawed outputs. Prejudice bias: Data from prejudiced sources can perpetuate systemic disparities. Cultural bias: Training data that reflects only certain segments of society can limit applicability. Cognitive bias: Influence by a few programmers can limit the applicability of outputs.
Bruna Marmett, 2018 [5]	Literature review conducted to explore the applications of artificial intelligence (AI) in the management of obesity	The review included 54 articles initially, with only seven meeting the selection criteria.	Individuals affected by obesity and healthcare professionals involved in their management	Public Medline (PubMed), Web of Science, Biblioteca Regional de Medicina (BIREME), and Google Scholar	Effectiveness of AI technologies in managing obesity and related health conditions	Independent variables include different AI systems and their specific applications in obesity management, such as decision support systems, MOPET app, parameter decreasing methods, artificial neural networks, neuro‐fuzzy models, image processing algorithms, and support vector machines.	Various algorithms were used across the different AI systems, including Bayesian networks, artificial neural networks, support vector machines, and image processing algorithms.	Sensitivity, specificity, accuracy, and error rates of the AI systems	The limited availability of literature specifically focusing on AI and obesity management The complex nature of AI systems, which makes them challenging to test and validate. The need for more advanced formal methods to check the reliability of AI software The potential for AI systems to provide inaccurate results if not properly designed and validated
Yuehjen E. Shao, 2014 [13]	Cross‐sectional design to develop and evaluate various forecasting models for predicting body fat percentage (BFP)	252	Individuals with body fat measurements determined by underwater weighing and 13 body circumference measurements	Real dataset obtained from Johnson (1996), which includes BFP measurements and various body circumference measurements	Body fat percentage (BFP), calculated using Siri's equation based on density determined from underwater weighing	13 Body circumference measurements: age, height, weight, neck circumference, chest circumference, abdomen 2 circumference, hip circumference, thigh circumference, knee circumference, ankle circumference, biceps (extended) circumference, forearm circumference, and wrist circumference	Multiple regression (MR) Artificial neural network (ANN) Multivariate adaptive regression splines (MARS) Support vector regression (SVR) Hybrid models: MR‐ANN, MR‐MARS, MR‐SVR, MARS‐MR, MARS‐ANN, and MARS‐SVR	Mean absolute percentage error (MAPE) Root mean square error (RMSE) Mean absolute difference (MAD)	Data limitations: The study relies on a single dataset with specific measurement methods, which may not generalize to other populations or measurement techniques. Variable selection: Although the study aims to reduce the number of explanatory variables, the selection process may exclude potentially important variables. Model complexity: Some hybrid models, despite their improved performance, may be more complex and require more computational resources compared to single‐stage models. Generalizability: The findings may not be directly applicable to all populations or conditions, and further validation is needed across diverse datasets.
Prakash Kn Bhanu, 2022 [20]	Prospective cohort study focusing on developing and evaluating a deep learning‐based tool for automated quantification of abdominal fat compartments from MRI images	190	Healthy older adults from the Geri‐LABS study	Geri‐LABS study	Accurate segmentation and quantification of abdominal fat compartments	Type of abdominal fat compartment (SSAT, DSAT, and VAT) and the characteristics of the MRI images, such as image contrast and quality	Standard 3D U‐Net: Used for initial segmentation of fat compartments Residual global aggregation‐based 3D U‐Net (RGA‐U‐Net): Enhanced version of the U‐Net with additional features for better segmentation accuracy	Dice coefficient, Hausdorff distance, correlation, and Bland–Altman analysis	Generalizability: The study's data were retrospectively derived from the Geri‐LABS cohort study, and the generalizability of the findings to other populations or datasets may be limited. Data variability: The study assumed that the MR scans were acquired under standardized conditions, which may not always be the case in practice. Ground truth variability: The ground truths were drawn by clinicians, and there may be variability in how different clinicians interpret and draw the boundaries of fat compartments. Technical reproducibility: The study did not evaluate the technical reproducibility of the MR scans, which could affect the consistency of the segmentation results. Overestimation and underestimation: There was some overestimation of VAT and underestimation of SSAT and DSAT in certain cases, which could be due to imaging errors or the complexity of the anatomical boundaries.
Seyed Taghi Heydari, 2012 [25]	Cross‐sectional study to compare the effectiveness of artificial neural networks (ANNs) and logistic regression in classifying obesity	414	Healthy military personnel from southern Iran with complete data on socioeconomic status and anthropometric measures	Data were collected through questionnaires and physical measurements by trained personnel.	Classification of participants as obese or nonobese	Demographic and lifestyle data: Age, marital status, level of education, duration of physical activity per week, history of smoking Anthropometric measures: weight, height, mid–upper arm circumference (MUAC), waist circumference (WC), hip circumference (HC), triceps skinfold thickness, and abdominal thickness	Logistic regression and artificial neural networks (ANNs)	Accuracy, sensitivity, specificity, area under the receiver‐operating characteristic (ROC) curve, and kappa statistic	Sample selection: The study was limited to healthy military personnel, which may not generalize to the broader population. Measurement methods: Although anthropometric measures are simple and noninvasive, they may not provide as accurate an assessment of obesity as more sophisticated methods like DXA or hydrostatic weighing. Model complexity: ANNs are considered “black box” models, making it difficult to interpret the relationships between input and output variables. Comparative analysis: The study found no significant difference between ANNs and logistic regression, which might limit the perceived benefit of using ANNs over traditional methods.
Tugba Barlas, 2024 [37]	Cross‐sectional study to assess the credibility of ChatGPT in the assessment of obesity in T2D according to current guidelines	20 Questions developed by experienced endocrinologists	ChatGPT‐3.5	Responses from ChatGPT‐3.5 to the 20 questions posed by endocrinologists	Credibility of ChatGPT's responses as evaluated against the latest American Diabetes Association (ADA) and American Association of Clinical Endocrinology (AACE) guidelines	The 20 questions covering assessment, nutrition, physical activity, behavioral therapy, pharmacotherapy, and medical devices and metabolic surgery	ChatGPT‐3.5	Compatibility with guidelines: Responses were categorized into four levels of compatibility with the guidelines: compatible, compatible but insufficient, insufficient, and incompatible. Understandability and comprehensiveness: Evaluated based on the clarity and completeness of the responses	Cross‐sectional nature: The study was conducted at a specific time, and AI programs update and improve over time. Subjectivity: The questions and assessments were partly subjective, despite being categorized independently by two experienced endocrinologists with discrepancies resolved by a blinded third senior reviewer. Specificity of AI version: The study analyzed answers from a specific version of ChatGPT to avoid confusion, which may not reflect the performance of other AI versions or models. Currency of information: ChatGPT‐3.5 was trained on data up to 2021, potentially limiting access to real‐time data or updates beyond its last training. Lack of transparency in data sources: The specifics of the datasets used to train ChatGPT, including whether proprietary medical databases were used, have not been publicly disclosed. Regional and personal variations: ChatGPT may not account for regional or personal variations in medical recommendations, which could be significant for patients.
Casimiro A. Curbelo Montaez, 2018 [49]	Case–control study design, utilizing deep learning to classify obesity based on genome‐wide association study (GWAS) single nucleotide polymorphisms (SNPs)	1997	Patients from the Geisinger Clinic with extreme obesity who have undergone bariatric surgery	Genotypes and Phenotypes (dbGaP)	Obesity, classified as a binary phenotype (obese vs. nonobese)	SNPs associated with obesity, selected based on *p* value thresholds from the GWAS analysis. Four subsets of SNPs were used: *p* values lower than 1 × 10^−5^ (5 SNPs), 1 × 10^−4^ (32 SNPs), 1 × 10^−3^ (248 SNPs), and 1 × 10^−2^ (2465 SNPs).	Deep learning framework, specifically a multilayer feedforward neural network trained with stochastic gradient descent using backpropagation and logistic regression	Sensitivity, specificity, Gini coefficient, logarithmic loss, area under the receiver operating characteristic curve (AUC), and mean squared error (MSE)	1. Deep learning model acts as a black box, making it difficult to interpret the results. 2. The study was limited to Caucasian individuals, which may not generalize to other ethnic groups. 3. The study relied on *p* value thresholds to select SNPs, which may not capture all relevant genetic variants.
Yang Xiao, 2020 [33]	Cross‐sectional design, utilizing both survey data and environmental data from Baidu Street View images	8988	Urban residents from 40 communities in Shanghai, China	The survey data on urban residents were obtained from the World Health Organization's Global Aging and Adult Health Data (Sage, Wave 1). The environmental data, including the View Green Index (VGI), NDVI, and park accessibility, were derived from Baidu Street View images and Landsat 8 satellite data.	Body mass index (BMI) categorized into three groups: normal, overweight, and obese	View green index (VGI), normalized difference vegetation index (NDVI), park accessibility, and demographic and socioeconomic variables such as age, gender, marital status, education level, income, employment status, and self‐rated health	Deep convolutional neural network and image segmentation and classification	Wald chi‐square test and the significance of the coefficients	Representativeness of the sample: The sample may not perfectly represent the overall greening condition of Shanghai, as it relies on data from specific communities and a survey that may not cover all population segments. Seasonal variability: The NDVI feature may vary across seasons, and the street view images used for the VGI are not season‐specific, which could affect the results. Uncertainty in street view data: The street view data are affected by the sampling distance and the quality of the street view images themselves, introducing potential uncertainties. Time of VGI data: The street view images are formed across the year, and it is difficult to identify the season of the images, making the time of VGI data uncertain.
Xiaolu Cheng, 2021 [52]	Cross‐sectional analysis to examine the relationship between physical activity and weight status and assess the performance and predictive power of a set of popular machine learning and traditional statistical methods	7162	Adults aged 20–85.	National Health and Nutrition Examination Survey (NHANES) 2003–2006	Body mass index (BMI) and weight status categories: normal weight (BMI 18.5 to < 25 kg/m^2^), overweight (BMI 25 to < 30 kg/m^2^), and obesity (BMI ≥ 30 kg/m^2^)	Physical activity (PA) levels: Sedentary, light, lifestyle, moderate, and vigorous Sociodemographic variables: gender, age, race, education level, marital status, and family poverty income ratio (PIR)	Logistic regression Naïve Bayes Radial basis function (RBF) Local *k*‐nearest neighbors (*k*‐NN) Classification via regression (CVR) Random subspace Decision table Multiobjective evolutionary fuzzy classifier Random tree J48 Multilayer perceptron	Overall accuracy, sensitivity, specificity, area under the receiver operating characteristic (ROC) curve (AUC)	Data limitations: The ActiGraph AM‐7164 device used for measuring physical activity is not waterproof, leading to potential underestimation of activities like swimming. Additionally, the device may not accurately detect upper‐body exercises. Scope of outcomes: The study focuses on weight status rather than body composition, which could provide more nuanced insights into obesity. Generalizability: The findings are based on a large, population‐based sample, but the generalizability to other populations or time periods may be limited. Complexity of machine learning models: Although machine learning models offer advanced predictive capabilities, their complexity can make them less interpretable compared to traditional statistical methods.
Sedat Arslan, 2023 [42]	The document does not specify a study design, as it primarily discusses the potential uses of Chat GPT in obesity treatment. It references various studies (e.g., chatbots for obesity counseling and personalized chatbots for obesity treatment) but does not detail a specific study design for the Chat GPT application.	No specific sample size is mentioned. The document discusses the general potential of Chat GPT in obesity treatment without referring to a particular study's sample size.	The document does not specify a study population. It broadly discusses the application of Chat GPT in obesity treatment but does not limit the discussion to a specific demographic or patient group.	The document does not provide details on the data source. It references the use of patient data for personalized recommendations but does not specify the origin or type of data used.	Effectiveness of obesity treatment, including weight management and the reduction of associated health risks.	Patient's medical history Physical characteristics Lifestyle factors Progress over time	The document mentions the use of Chat GPT. It does not specify other algorithms used for predictive modeling or data analysis.	The document does not provide specific model performance metrics. It discusses the potential effectiveness of Chat GPT in providing personalized recommendations and predictive modeling but does not present quantitative performance measures.	Ethical implications: The document discusses the ethical considerations of relying on AI for healthcare decisions and the potential bias in training data. Data security: Concerns are raised about the security and privacy of patient data. Emotional intelligence: Chat GPT lacks true emotional intelligence and may not provide the same level of emotional support as human healthcare providers. Technical limitations: The resource‐intensive nature of GPT models and the requirement for high‐speed internet or advanced computing technology may limit their use in certain settings. Accountability: In cases of incorrect or harmful advice, there may be questions about accountability, as AI models do not adhere to professional standards or ethics codes.
David Scheinker, 2019 [43]	Cross‐sectional study using linear regression models and machine learning models.	3138	All counties within the United States, representing a broad geographic and demographic spectrum	2018 Robert Wood Johnson Foundation County Health Rankings (CHR) and US Census data	County‐level obesity prevalence	Demographic factors: population, rural status, census region, race/ethnicity, sex, and age composition Socioeconomic factors: median income, unemployment rate, and percentage of the population with some college education	Univariate and multivariate linear regression; gradient boosting machine (GBM), regression trees, random forest, linear models selected using Akaike information criterion (AIC), Bayesian information criterion (BIC), and their variants, as well as penalized linear models chosen using elastic net variants of the least absolute shrinkage and selection operator (LASSO)	*R* ^2^ (coefficient of determination)	Data quality and source: The study relies on self‐reported and interpolated data, which may introduce bias and affect the accuracy of the findings. Self‐reported bias: Obesity prevalence is based on self‐reported height and weight, which may underestimate actual obesity rates. Generalizability: The study's findings are limited to the variables captured in the datasets used and may not account for individual‐level risk factors. Interpretability of machine learning models: Although machine learning models offer improved performance, they are less interpretable than traditional regression models, making it harder to understand the specific relationships between variables. BMI as a measure: The study uses BMI as a proxy for obesity, which may not accurately reflect adiposity and health risks across different demographic groups.
Ayan Chatterjee, 2020 [39]	Systematic review and meta‐analysis of scientific literature, followed by a statistical analysis using machine learning (ML) models focusing on identifying risk factors associated with obesity/overweight and understanding their correlation with weight change	Insurance dataset: 1338 BMI‐related dataset: 500 Eating‐health‐module dataset: 11,212	Adults aged between 20 and 60, excluding pregnancy and genetic factors	Kaggle, UCI	Obesity/overweight Weight change Cardiovascular diseases (CVDs) Type 2 diabetes	Age Sex Body mass index (BMI) Height Weight Tobacco consumption Consumption of sweet beverages Economic condition Fast food consumption Sleeping pattern Diet Blood pressure Blood glucose level Lipid profile Adiposity Exercise Family history	Support vector machine (SVM) naïve Bayes Decision tree Logistic regression *k*‐nearest neighbors (*k*‐NN) Random forest (RF) linear regression *k*‐nearest neighbor regressor Support vector regressor Decision tree regressor Random forest regressor Bayesian regressor Lasso regression (L1) Ridge regression (L2)	Accuracy, *k*‐fold cross‐validation, and Brier score metric for probability calibration	The study does not have a single dataset that combines all intended risk factors. The data from different sources are not from the same target population, making it difficult to combine them into a single source. Small dataset sizes after removing heterogeneous features may not be appropriate for ML model training with cross‐validation. The study excludes genetic factors and pregnancy, which could be significant risk factors for obesity/overweight. The future research plan includes collecting data from south Norway, which may introduce a regional bias.
Philippe J. Giabbanelli, 2024 [48]	Participatory modeling approach combined with artificial intelligence techniques, specifically network science and natural language processing that aims to understand the factors influencing obesity and well‐being and the implications for public policymaking. The design involves semistructured interviews with experts to elicit knowledge, which is then structured into a conceptual map.	19	Participants were chosen for their expertise in various domains relevant to the study, including physical well‐being, mental well‐being, and obesity.	Collected through semistructured interviews with the selected experts	Shift from an obesity‐centric to a well‐being‐centric paradigm in public health	Availability of healthy food options Built environment (pollution level, aesthetics, infrastructure) Community ties Culture of eating healthy food Eating behavior Economy Exercise Family dynamics Income Perceptions of neighborhood safety Political climate Public health messaging School environment Work environment	For network analysis include node centrality algorithms, as well as for natural language processing, standard text analysis procedures such as stemming and removal of common English words were applied.	The document does not explicitly mention specific performance metrics for the model. However, it does discuss the use of network analysis to identify critical components within the interconnected system, which can provide insights for public health interventions.	The study is limited by the perspectives of its participants, as participatory modeling reflects the views of those involved in the study. The study did not include individuals with lived experiences of overweight and obesity, which could offer complementary perspectives. The network analysis was guided by the focus on a proposed paradigm shift from obesity to well‐being, and other potential analyses were not explored in the current study. The map is a comprehensive model spanning multiple domains, and space limitations precluded its inclusion in the main body of the paper.
Mathias J. Gerl, 2019 [16]	Large‐scale observational analysis that utilizes machine learning techniques to estimate obesity measures such as body fat percentage (BFP), body mass index (BMI), waist circumference (WC), and waist–hip ratio (WHR) using plasma lipidome data. The study design includes a comprehensive analysis of lipid species in plasma samples from a large population cohort, combined with advanced machine learning modeling.	1061 Participants from the FINRISK 2012 cohort and 250 participants from the Malmö Diet and Cancer Cardiovascular Cohort (MDC‐CC)	FINRISK 2012 cohort and the MDC‐CC	FINRISK 2012 cohort and the MDC‐CC	Body fat percentage (BFP), body mass index (BMI), waist circumference (WC), and waist–hip ratio (WHR)	Plasma lipidome data, age, and sex. The lipidome data are detailed and includes 183 lipid species, which are used as predictors in the machine learning models.	Lasso (least absolute shrinkage and selection operator) Random forest Stochastic gradient boosting Partial least squares Cubist	Mean absolute error (MAE) Normalized mean absolute error (NMAE) Coefficient of determination (*R* ^2^)	Although the document does not explicitly list the limitations, common limitations in such studies could include the following: the generalizability of the findings to other populations or cohorts potential confounding factors not accounted for in the analysis, the complexity and interpretability of the machine learning models, the need for validation in independent cohorts to confirm the predictive accuracy of the models, and the cost and feasibility of implementing lipidomic profiling in routine clinical setting.
Ziwei Lin, 2021 [29]	Multicenter, retrospective study to identify metabolic subtypes of obesity using machine learning	2495	Patient with obesity from Shanghai Tenth People's Hospital and three additional cohorts from different hospitals in China	Shanghai Tenth People's Hospital and three additional cohorts from different hospitals in China	Identification of metabolic subtypes of obesity	AUCs of glucose and insulin during oral glucose tolerance test (OGTT) and uric acid levels	Clustering algorithms: *k*‐means and two‐step clustering	Accuracy Jaccard similarity coefficient	Generalizability: All patients are of Chinese ethnicity, limiting the applicability to other ethnicities. Retrospective design: Potential differences in measurements and lab tests across institutions. Data imputation: Some data points were missing, requiring imputation, which may introduce inaccuracies. Proof of concept: Further validation in a prospective setting is needed to confirm the clinical value of the identified subtypes.
Vicent Blanes‐Selva, 2020 [28]	Machine learning‐based approach, specifically positive and unlabelled (PU) learning, to identify obese patients within a hospital setting	49,805	Patients from the Hospital Universitario La Fe in Valencia, Spain.	Electronic health records (EHRs) of the Hospital Universitario La Fe	Identification of obese patients	32 Predictive variables, including diagnoses, specialist consultations, and laboratory results	Implementation of the “Bagging Inductive PU Learning” algorithm proposed by Mordelet et al.	Sensitivity and the estimation of negatives in the unlabelled set	The potential bias toward ignoring obese patients who have not utilized hospital resources extensively. The study's findings may not generalize to populations outside the Hospital Universitario La Fe or to settings with different healthcare practices and data recording methods.
Fabio Buttussi, 2008 [30]	Introduces MOPET, a wearable system designed to enhance physical fitness training through context‐aware and user‐adaptive features	12	Individuals seeking to improve their fitness and health through personalized training support	Real‐time data from sensors such as a heart rate monitor, a 3D accelerometer, and a PDA with GPS. Additionally, the system uses expert knowledge from a sport physiologist and a professional trainer, and a user model updated via a guided autotest.	Improvement in user interaction and training effectiveness	Personal information (weight, height, gender, and age) Physiological information (maximum volume of oxygen the user can consume in a minute) User's experience with each strengthening exercise Real‐time sensor data (heart rate, position, and exercising time)	The study does not explicitly mention specific algorithms, but it implies the use of context‐aware and user‐adaptive algorithms to analyze sensor data and provide personalized training advice.	The performance metrics are not explicitly mentioned, but the effectiveness of MOPET is demonstrated through its ability to guide users through autotests, provide real‐time jogging support, and offer context‐aware exercise advice.	The study does not specify the duration of the prototype evaluation or the long‐term effects of using MOPET. The sample size of 12 users may be considered small, and the results might not be generalizable to a larger population. The study does not discuss the potential technical limitations or challenges in integrating various sensors and ensuring the accuracy of the data. Future research directions include enhancing adaptation capabilities, integrating additional sensors, and developing offline data analysis tools, indicating that the current system has room for improvement.
Yumin Yao, 2020 [51]	Cross‐sectional analysis using two public datasets to evaluate the proposed hybrid deep neural network model for predicting body mass index (BMI) from smartphone motion sensor data	MobiAct: 67; Motion‐Sense: 24	Individuals whose motion sensor data were recorded using smartphones during various activities of daily living (ADLs) such as jogging, walking, and walking upstairs	MobiAct and Motion‐Sense	Body mass index (BMI)	Motion sensor data collected from the smartphones, including accelerometer and gyroscope readings	Hybrid deep neural network model that combines convolutional neural networks (CNNs) and long short‐term memory (LSTM) networks. Additionally, a novel motion entropy (MEn)–based filtering strategy is used to preprocess the sensor data.	Mean absolute error (MAE) and root mean square error (RMSE)	Dependence on labeled data: The performance of the deep learning model heavily relies on the availability of labeled data, which can be expensive and time‐consuming to obtain. Generalizability: The study's findings may not generalize to all populations, as the datasets used were collected from specific demographics (mainly university campuses). Activity labels: The study requires activity labels for training the model. Developing a method for predicting BMI from sensor data without activity labels (unsupervised human activity recognition) remains a challenge. Privacy concerns: Although motion sensors are considered less privacy‐sensitive, there are still concerns regarding the collection and analysis of personal data. Limited data for extreme BMI categories: The datasets used in the study have limited samples for underweight and obesity categories, which may affect the model's accuracy in predicting BMI for these groups.
Angela K. Fitch, 2022 [35]	A compilation of best practices and recommendations based on published scientific literature and clinical perspectives of OMA authors, which is designed to provide clinicians with organizational tools and guidelines for managing obesity	Not applicable as this is not a research study with a defined sample size	The CPS is intended for clinicians and healthcare professionals involved in the management of obesity	Based on published scientific citations, clinical perspectives of OMA authors, and peer review by Obesity Medicine Association leadership	Improve the understanding and management of obesity among clinicians. It does not specify a traditional outcome variable as seen in research studies.	Not applicable in the context of a CPS. The document provides guidelines rather than testing specific variables.	The CPS references the OMA's “ADAPT” method for telehealth obesity management, which includes assessment, diagnosis, advice, prognosis, and treatment. This method serves as a structured approach to managing obesity in virtual settings.	As this is not a predictive model, performance metrics like accuracy, sensitivity, specificity, or area under the curve (AUC) do not apply.	The document itself does not list specific limitations. However, general limitations of such guidelines include the potential for variation in interpretation and application across different clinical settings, the need for ongoing updates as new evidence emerges, and the challenges of addressing individual patient variability within standardized guidelines.
Mehak Gupta, 2022 [53]	Deep learning approach using long short‐term memory (LSTM) networks to predict childhood obesity	68,003 Unique patients and 3,496,559 distinct visits	Children and adolescents aged 2–20 years	Nemours Children Health System	Obesity status (obese vs. nonobese)	Clinical and demographic data extracted from the EHR system. These variables encompass condition codes, medication codes, procedure codes, measurement codes (lab results), and demographic information such as sex, race, ethnicity, and zip code	Deep learning model based on LSTM networks and transfer learning	Accuracy, area under the receiver operating characteristic curve (AUC), sensitivity, and positive predictive value (PPV)	The model can predict a maximum of 3 years into the future because of the requirement of at least 5 years of medical history data for each patient. The features in the EHR data are not grouped, leading to a large number of variables, most of which are not recorded for a significant portion of the population. The current feature‐level interpretability process includes correlated features.
Ramyaa Ramyaa, 2019 [32]	Retrospective analysis that employs machine learning algorithms to predict body weight and identify phenotypes based on dietary macronutrient intake, physical activity, and sociodemographic variables	63,060	Postmenopausal women enrolled in the WHI OS	Biological Specimen and Data Repository Information Coordinating Center (BioLINCC), which hosts the WHI OS data	Body weight, both in terms of numerical prediction (in kilograms) and categorical prediction (as body mass index (BMI) categories)	Dietary macronutrients (carbohydrates, proteins, fats, fibers, sugars, and alcohol), physical activity levels (mild, moderate, and vigorous intensity), sociodemographic variables (age, ethnicity, socioeconomic score, and marital status), and health status (presence of diseases such as diabetes, and hypertension)	Support vector machines (SVMs) Neural networks *k*‐nearest neighbors (*k*‐NN) Decision trees Ensemble methods (bagged trees, AdaBoost) *k*‐means clustering	Mean approximate error (MAE) *R* ^2^ Root mean square error (RMSE) Accuracy	Self‐reported data: The data are based on self‐reported dietary intake and physical activity, which are prone to bias and errors. Model fit: Although the overall data model showed a reasonable fit, the modest performance suggests variability and noise in the data, which may limit the generalizability of the findings. Underreporting: There is evidence of underreporting in dietary data, particularly among overweight and obese individuals, which may affect model accuracy. Sample size and representation: Although the sample size is large, the distribution of data across different BMI categories may not be adequate for robust model building, especially for underweight and obese categories. Complexity vs. simplicity: The study found that simpler models like *k*‐NN performed comparably to more complex models, suggesting that the data may inherently satisfy the assumptions of simpler methods. Clustering limitations: Although clustering improved prediction accuracy, the ability to predict the cluster for new cases was not always reliable, indicating limitations in the classifier model or data quality.
Sri Astuti Thamrin, 2021 [18]	Cross‐sectional analysis for the examination of the prevalence of obesity and associated risk factors at a single point in time	634,709 respondents, with 618,898 records	Adults aged 18 and above from across Indonesia	RISKESDAS survey conducted by the Indonesian Ministry of Health	Obesity status, determined by the body mass index (BMI) of the respondents	Location Marital status Age group Education Work category Sugary foods Sweet drinks Salty foods Fatty/oily foods Grilled foods Preserved foods Seasoning powders Soft/carbonated drinks Energy drinks Instant foods Alcoholic drinks Mental–emotional disorders Diagnosed hypertension Physical activity Smoking Fruit and vegetable consumptions	Logistic regression Classification and regression trees (CART) naïve Bayes	Accuracy Sensitivity Specificity Precision Recall F1 score Kappa Area under the curve (AUC)	The study is based on cross‐sectional data, which limits the ability to establish causal relationships. Some relevant factors such as sex, dietary quality, clinical and physiological factors, wealth, and genetic and cultural influences were not included, potentially affecting the results. The RISKESDAS data are collected every 5 years, which may not capture the most recent trends or rapid changes in obesity prevalence and risk factors. The study's findings are based on Indonesian data and may not be generalizable to other populations.
Rajdeep Kaur, 2022 [21]	Retrospective analysis using machine learning (ML) algorithms to predict obesity levels and provide personalized meal planning	2111	Individuals with varying physical descriptions and eating habits, representing a diverse group of people with different obesity levels	Obesity prediction dataset: UCI ML repository. Meal planning dataset: open‐source websites	Obesity level, categorized into several classes including underweight, normal weight, overweight, and obesity (Type I, Type II, and Type III)	Physical description features: age, height, weight, gender, family history of overweight. Eating habit features: Frequency of main meals, frequency of consumption of high‐calorie meals, frequency of consumption of vegetables, smoking, consumption of alcohol, and daily water intake	Gradient boosting (GB) Bagging meta‐estimator (BME) XG Boost (XGB) Random forest (RF) Support vector machine (SVM) *k*‐nearest neighbor (*k*‐NN)	Accuracy Precision Recall F1 score	Data limitations: The study relies on data from specific sources, which may not fully represent the global population. Algorithmic limitations: Although the study uses several ML algorithms, there may be other advanced techniques that could improve prediction accuracy. Generalizability: The models may need further validation to ensure they are generalizable to different populations and datasets. Physical activity: The study does not consider physical activity data, which is a significant factor in obesity management. Meal dataset: The meal planning dataset is limited in size and variety, which may affect the robustness of the meal recommendations. Cultural and personal preferences: The study does not account for individual cultural, seasonal, or personal food preferences, which can significantly influence meal planning.
Han Shi Jocelyn Chew, 2024 [41]	Cross‐sectional study to examine the public acceptance and intention to use AI‐assisted weight management apps among high‐income Southeast Asian adults with overweight and obesity	271	Participants completed a questionnaire on sociodemographic profiles, Unified Theory of Acceptance and Use of Technology 2 (UTAUT2), and Self‐Regulation of Eating Behavior Questionnaire	Collection method: online survey Components: sociodemographic profile, UTAUT2 questionnaire, and Self‐Regulation of Eating Behavior Questionnaire (SREBQ)	Intention to use AI‐assisted weight management apps	Performance expectancy Effort expectancy Social influence Facilitating conditions Hedonic motivation Price value Habit Other variables: age, anxiety risk, intention to have a healthy diet, self‐regulation, depression risk, BMI, and waist circumference	Two‐stage SEM approach, combining factor analysis and multiple regression analysis	Model chi‐square Comparative fit index (CFI) Root mean square error of approximation (RMSEA) Standardized root mean square residual (SRMR)	Generalizability: The study's findings may not be generalizable to populations outside of high‐income Southeast Asian adults with overweight and obesity. Self‐reported data: The data on BMI and waist circumference were self‐reported, which could introduce measurement errors. Common method bias: Although efforts were made to mitigate common method bias, the potential for such biases cannot be entirely ruled out. AI app experience: The study did not specify the level of participants' experience with AI‐assisted apps, which could influence their intention to use such apps.
Abdulqadir J. Nashwan, 2024 [38]	Not applicable as this is an editorial discussing the potential of AI in obesity management	Not applicable	Not applicable	The editorial discusses the use of AI to analyze various types of data, including genetic, lifestyle, and medical data, but it does not specify a particular data source	Improvement of weight management strategies and the shift toward precision medicine	The editorial mentions that AI can consider various factors such as genetic information, medical history, lifestyle factors, and dietary habits, but it does not provide specific independent variables.	The editorial does not specify particular algorithms but discusses the use of AI in general.	Not applicable as specific models or their performance metrics are not discussed.	Not applicable as specific models or their performance metrics are not discussed.
Antonio Ferreras, 2023 [26]	Systematic review that employs the Preferred Reporting Items for Systematic Reviews and Meta‐Analyses (PRISMA) protocol aiming to identify, analyze, and evaluate research articles that apply machine learning (ML) and deep learning (DL) models to the prediction of obesity and overweight	17 Articles that were selected after applying the PRISMA protocol	Research articles that focus on the application of ML and DL models to predict obesity and overweight. The population is not limited to a specific age group or geographic location but encompasses studies that have utilized these models in the context of obesity and overweight prediction.	The data sources for the study include various academic search engines such as Google Scholar, PubMed, IEEE Xplore, and ScienceDirect.	Prediction of obesity and overweight	The different ML and DL models, algorithms, and the conditions under which these models are applied	Traditional ML models like decision trees (DTs), random forests (RFs), support vector machines (SVMs), and Bayesian networks (BNs), as well as DL models like multilayered feedforward neural networks (MLFNN), long short‐term memory (LSTM) networks, and others	Accuracy, sensitivity, specificity, area under the curve (AUC), and other relevant statistical measures	One of the main limitations of the study is that it only includes articles published in English between January 1, 2015, and June 2022. This means that articles published before 2015 or in other languages are not included. Additionally, the review does not include an evaluation of bias, which is a common component in systematic reviews. Despite these limitations, the study provides a comprehensive overview of the current state of research on ML and DL in the prediction of obesity and overweight.
Uçman Ergün, 2009 [12]	Cross‐sectional design and comparative analysis of logistic regression and neural network methods for classifying obesity	82	Participants were selected based on specific criteria related to their health status and body mass index (BMI)	The data were collected from 82 individuals.	Classification of individuals as either obese or healthy	24 Independent variables such as BMI, TA/systole, TA/diastole, liver right lobe, vena porta, RCI, RCC, RCCAmax, RCCAmin, RICAmax, RICAmin, LCI, LCCA, LCCAmax, LCCAmin, LICAmax, LICAmin, CTR, aorta knot, hilus big, hilus down, diagraph down, and lung arbre	Logistic regression Neural network	Sensitivity, specificity, and area under the ROC curve	The study may have limitations in terms of the generalizability of the results, as the sample size is relatively small and may not fully represent the broader population. The study focuses on a specific set of parameters and may not account for other factors that could influence obesity classification. The performance metrics, although promising, may still leave room for improvement, indicating potential areas for further research and model optimization.
Nigel Hinchliffe, 2022 [40]	Review paper synthesizes findings from various studies, including systematic reviews, meta‐analyses, and case studies, to provide an overview of the current state of digital health in obesity care.	As a review, it does not have a sample size.	The review covers studies that include patients with obesity and healthcare providers involved in obesity care.	The data sources include a wide range of studies, systematic reviews, and meta‐analyses from the existing literature.	The primary outcome variables discussed in the review include weight loss, weight management, physical activity, dietary adherence, and overall health outcomes related to obesity.	The review discusses various factors that influence the effectiveness of digital health interventions, such as the type of technology used (telehealth, mHealth apps, and wearable devices), duration of interventions, frequency of use, and the presence of personalized features.	The review highlights the use of machine learning and artificial intelligence algorithms in personalizing obesity interventions. Specific algorithms mentioned include decision trees, artificial neural networks, and predictive analytics for behavior change.	The review does not provide specific model performance metrics as it aggregates findings from multiple studies. Individual studies may have used metrics such as accuracy, precision, recall, F1 score, and area under the curve (AUC) to evaluate the performance of their models.	The review acknowledges several limitations, including the heterogeneity of studies, potential biases in the literature, and the need for more research to establish best practices and identify successful patient phenotypes for remote obesity care. It also discusses the challenges of ensuring equitable access to digital health technologies and the need for further research into the long‐term effectiveness of these interventions.
Giovanni Delnevo, 2021 [19]	Confirmatory research design, utilizing machine learning (ML) algorithms to predict body mass index (BMI) values and status based on psychological variables	221	Adults seeking treatment for obesity and a control group. Participants were organized into three BMI categories groups: normal weight, overweight, and obese	The data used in this study were psychological variables collected from adults seeking treatment for obesity and a control group.	Body mass index (BMI), which was considered both as a continuous variable (BMI values) and a categorical variable (BMI classes: normal weight, overweight, and obese)	Psychological variables, including both positive (e.g., trait emotional intelligence, cognitive reappraisal, and happiness) and negative (e.g., depression, trait and state anxiety, and binge eating) variables	*k*‐Nearest neighbor (*k*‐NN) Classification and regression tree (CART) Support vector machine (SVM) Multilayer perceptron (MLP) Adaptive boosting with decision tree (AB) Gradient boosting (GB) Random forest (RF) Extra tree (ET) Lasso regression Elastic net regression	For classification: F1 score, sensitivity, and specificity For regression: mean absolute error (MAE) and Pearson correlation coefficient (PCC)	The study did not employ newly collected data, which limits the inferences that can be drawn. The cross‐sectional design and reliance on self‐evaluation for most variables (except BMI values) are methodological limitations. The dataset size is restricted, which may limit the generalizability of the findings and the use of more complex algorithms. The study considered only psychological and demographic variables, without including medical or lifestyle factors that could contribute to the explanation of the results.
Diana M. Thomas, 2024 [44]	The review does not have a specific study design as it is a literature review discussing the transformation of Big Data into AI‐ready data for nutrition and obesity research. It covers the preprocessing steps, challenges, and opportunities for advances in this area.	Not applicable to a review article	Not applicable to a review article	The review discusses three major Big Data sources: microbiome, metabolomics, and accelerometry. It highlights the preprocessing steps and challenges associated with each data source	The review does not have a specific outcome variable. Instead, it discusses the transformation of raw data into AI‐ready datasets and the impact of preprocessing decisions on the final data quality and utility.	Not applicable to a review article	The review mentions the use of machine learning (ML) and artificial intelligence (AI) algorithms in the preprocessing of Big Data. It discusses the importance of human judgment and specialized software in this process.	Not applicable to a review article	The review highlights several challenges and limitations in the preprocessing of Big Data for nutrition and obesity research, including the complexity of the preprocessing steps, the need for human judgment, and the variability in data quality and veracity. It also discusses the lack of standardization in preprocessing approaches and the need for improved methods and tools to streamline the process.
Mahin Khan Mahadi, 2024 [23]	Comprehensive analysis of obesity levels using supervised machine learning models, incorporating explainable artificial intelligence (XAI) for model interpretability	2111	Individuals from three Latin American countries: Mexico, Peru, and Colombia	UCI machine learning repository	Obesity level, categorized into seven possible categories: insufficient weight, normal weight, overweight level I, overweight level II, obesity type I, obesity type II, and obesity type III	Attributes describing individuals' eating habits and physical condition, such as age, height, weight, frequency of consumption of different food groups, number of main meals per day, amount of water intake, level of physical activity, body mass index, and weight	Label propagation (LP), label spreading (LS), *k*‐nearest neighbor classifier (KNC), extra trees classifier, linear SVC, random forest (RF), extra tree classifier (ETC), bagging classifier (BC), decision tree classifier (DT), linear discriminant analysis (LDA), support vector classifier (SVC), NuSVC, logistic regression (LR), extreme gradient boosting (XGB), and light gradient boosting machine (LGBM)	Accuracy Weighted precision Weighted recall Weighted F1 score	Dataset imbalance: The dataset includes both synthetic and real data, which might introduce biases. Generalizability: The findings may not be generalizable to populations outside the three Latin American countries studied. Hyperparameter tuning impact: Although hyperparameter tuning was performed, its impact on model performance was limited, suggesting the need for further optimization. Feature selection: The study could benefit from a more detailed feature selection process to identify the most relevant variables. Model interpretability: Although XAI was used, there is room for improvement in making the models more interpretable for nonexperts.
Adyasha Maharana, 2018 [15]	Cross‐sectional design to examine the association of the built environment with prevalence of neighborhood adult obesity	150,000 High‐resolution satellite images and obesity prevalence data from 1695 census tracts	Adult residents in the selected urban areas, with obesity prevalence data obtained from the Centers for Disease Control and Prevention's 500 Cities project	Built environment data: High‐resolution satellite images from Google Static Maps API. Obesity prevalence data: From the Centers for Disease Control and Prevention's 500 Cities project	Obesity prevalence, defined as the percentage of adults with a body mass index (BMI) greater than or equal to 30	Features of the built environment extracted from satellite images using convolutional neural networks (CNNs), including the presence of parks, highways, green streets, crosswalks, and diverse housing types	Convolutional neural networks (CNNs): Specifically, the VGG‐CNN‐F network was used to extract features from satellite images. Elastic net regression: Applied to build a parsimonious model to assess the association between the built environment and obesity prevalence	*R*‐squared (*R* ^2^) Root mean square error (RMSE)	Data limitations: The obesity prevalence estimates were based on self‐reported height and weight, which can be biased. Additionally, the study used data from 2014 because of the unavailability of more recent estimates. Interpretability of features: The CNN used was trained for object recognition, which may limit the interpretability of the features used in the model. Temporal disparities: There were differences in the timing of the obesity data and satellite images, potentially introducing bias. Generalizability: The findings may not be generalizable to all geographic regions or demographic groups. Potential confounding factors: Although the study controlled for certain variables, other unmeasured factors may have influenced the results.
Nigmet Koklu, 2024 [24]	Cross‐sectional design, utilizing an online survey to collect data from participants to gather information on various factors related to obesity, including social and physical activities	1610	Individuals from Türkiye who participated in the online survey	Online questionnaire administered to participants living in Türkiye	Classification of obesity status, which included four categories: underweight, normal, overweight, and obesity	Sex Age Height Overweight/obese families Consumption of fast food Frequency of consuming vegetables Number of main meals daily Food intake between meals Smoking Liquid intake daily Calculation of calorie intake Physical exercise Schedule dedicated to technology Type of transportation used	Artificial neural network (ANN) *k*‐nearest neighbors (*k*‐NN) Random forest (RF) Support vector machine (SVM)	Accuracy (AC) Precision (P) Recall (R) F1 score (F)	The study relied on self‐reported data, which may introduce biases due to inaccuracies in self‐reporting. The dataset may not be fully representative of the general population, as it was limited to individuals from Türkiye who participated in the online survey. The study did not account for potential confounding variables that could affect the obesity status, such as underlying medical conditions or genetic factors. The classification success rates varied among the different algorithms, indicating that although some models performed well, there is still room for improvement in predicting obesity status. The study did not explore the impact of different types of physical activities on obesity, which could provide more nuanced insights into the relationship between physical activity and obesity.
Huiling Chen, 2015 [14]	Cross‐sectional analysis using a machine learning approach to predict overweight status based on blood and biochemical indexes	476	The study population consisted of both males and females, with a total of 179 males and 297 females. The age range and specific demographics were not detailed in the summary.	The First Affiliated Hospital of Wenzhou Medical University	Overweight status	18 Blood indexes and 16 biochemical indexes, such as triglyceride (TG), glucose (GLU), low‐density lipoprotein (LDL), high‐density lipoprotein (HDL), total cholesterol (CHO), alanine transaminase (ALT), aspartate aminotransferase (AST), γ‐glutamyl transpeptidase (γ‐GT), total protein (TP), albumin (ALB), creatinine (CR), urea nitrogen (BUN), alkaline phosphatase (AKP), total bilirubin (TBIL), direct bilirubin (DBIL), uric acid (UA), and others	Extreme learning machine (ELM) Support vector machines (SVMs) Backpropagation neural network (BPNN): Also used for comparison	Classification accuracy (ACC) Area under the receiver operating characteristic curve (AUC) Sensitivity Specificity	Sample size: The number of overweight subjects (225) may be considered relatively small, which could affect the generalizability of the results. Data source: The use of retrospective data from a single hospital may introduce bias and limit the diversity of the sample. Feature selection: Although the study used the Fisher score for feature selection, other methods could yield different results, potentially influencing the model's performance. Model generalization: The study's findings need to be validated with larger and more diverse datasets to ensure the model's generalizability across different populations. Potential confounding variables: The study did not account for potential confounding variables such as diet, physical activity, and underlying health conditions that could affect the blood indexes. BMI as a gold standard: The reliance on BMI as the sole criterion for overweight status may not capture the full complexity of body composition and health risks.
Ben Allen, 2022 [46]	Cross‐sectional design focusing on identifying interactions between environmental, social, and individual factors that contribute to obesity in adolescents	11,112	Adolescents aged 9–10 years from 22 sites across the United States	Data were collected as part of the ABCD study (Release 3.0.1), which includes baseline data and dietary intake data from the 1‐year follow‐up visit.	Age and sex‐adjusted waist‐to‐height ratio *z*‐scores, used as a measure of obesity	20 Features as independent variables, categorized into intrapersonal (e.g., pubertal stage, nutrition, and physical activity), interpersonal (e.g., family history and parent demographics), and community (e.g., crime, pollution, and poverty) levels	Random forest	Median variance	Cross‐sectional design: The study's cross‐sectional design limits the ability to make causal claims about the discovered interactions and obesity. Observational data: The reliance on observational data means that the study cannot control for potential confounding variables or establish causation. Census‐based proxies: The use of census‐based proxies for neighborhood boundaries may not accurately reflect the geographical distribution of causal factors linked to obesity. Aggregated data: The study uses aggregated data for neighborhood factors, which may not capture the heterogeneity within communities. Lack of mechanism definition: The study does not define the mechanisms for the discovered interactions, which are necessary for informing public policy changes.

**FIGURE 2:**
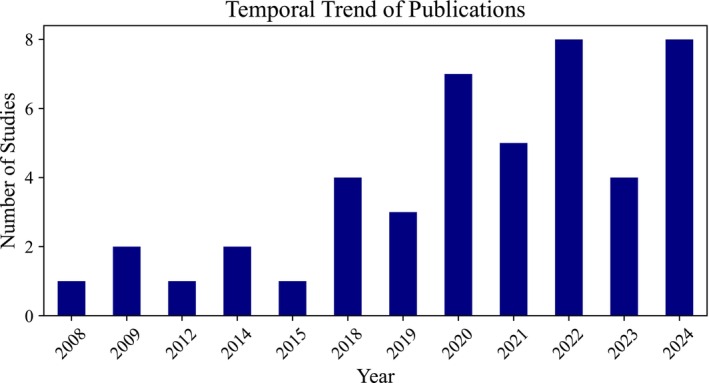
Temporal trend of publications.

Figure 2 illustrates the annual number of articles from 2008 to 2024 on the applications of AI in obesity prevention. The data shows a sparse distribution in the early years, with only one study in 2008 and a slight increase in 2009. A notable rise began around 2018, coinciding with growing interest in AI‐driven health solutions. The upward peak in 2020, with seven studies, highlights increased research activity, likely driven by advancements in AI technologies such as machine learning and neural networks applied to obesity prevention. Although a slight dip was observed in 2021, the trend continues upward, reaching another peak in 2022. The years 2023 and 2024 continued to show strong publication activity, indicating sustained research interest and momentum in AI applications for adult obesity. This trend highlights the evolving focus on integrating AI into healthcare, showcasing significant developments and progress in both technological and medical research fields.

**FIGURE 3:**
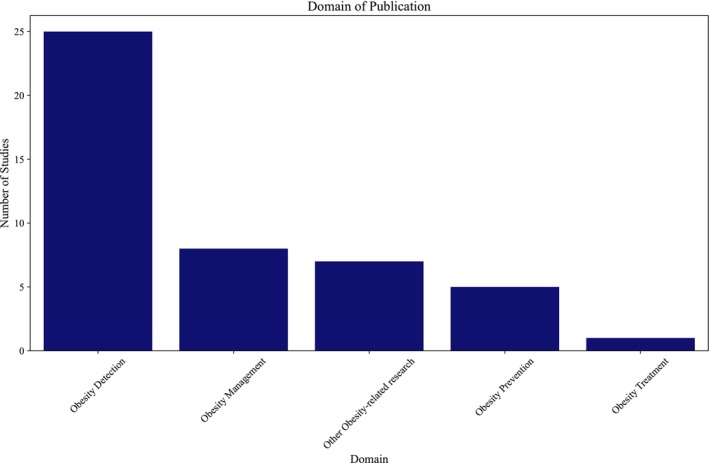
Domain of analysis.

Figure 3 presents a domain analysis of studies included in this systematic review on AI applications in obesity prevention, categorizing the research into five areas: obesity detection, management, treatment, prevention, and other obesity‐related research. The “obesity detection” category stands out with the highest number of studies, indicating a strong focus on identifying obesity. In contrast, the domains of prevention, management, and treatment have fewer studies, suggesting these are emerging fields for AI applications in obesity. This distribution highlights opportunities and the need for more targeted research in AI‐driven strategies for obesity prevention, management, and treatment.

The key findings from the reviewed articles indicate a multifaceted approach to understanding obesity and its related factors. The primary outcome variables studied include obesity/overweight, weight change, cardiovascular diseases (CVDs), and Type 2 diabetes, with a focus on shifting from an obesity‐centric to a well‐being‐centric paradigm in public health.

Several independent variables were identified, including age, sex, body mass index (BMI), tobacco consumption, and dietary habits, which were found to influence obesity and overall well‐being. Machine learning algorithms, including SVMs, random forest, and logistic regression, were employed to analyze the data, with model performance evaluated using metrics like accuracy, precision, and recall. Limitations included potential biases in self‐reported data, the need for more detailed feature selection, and challenges in generalizability due to the diversity of the sample.

This systematic review highlights that most AI applications in obesity research are heavily focused on detection, primarily employing supervised learning techniques, as shown in Table 2. This suggests that detection methodologies are more advanced than those in other areas of obesity research. There is notable variability in the methodological approaches, with diverse testing strategies, such as *k*‐fold cross‐validation, being commonly used. However, the lack of detailed reporting on calibration methods highlights a need for more rigorous and standardized reporting practices. The frequent tuning of hyperparameters underscores a strong focus on optimizing algorithmic performance, reflecting a commitment to improving model accuracy and reliability.

**TABLE 2:** Technical characteristics of the studies reviewed.

Machine learning algorithms used	Domain of applications	Number of studies	Testing strategies	Number of studies	Calibration approaches used	Number of studies	Hyperparameters adjusted	Number of studies	Whether models/code are shared	Number of studies
Supervised learning (Total: 27)	Obesity detection	19	*k*‐Fold cross‐validation	9	Not explicitly mentioned	16	Adjustments and tuning	8	Not indicated	16
	Another obesity‐related research	3	Not mentioned	5	Performance metrics	5	Not discussed	8	Available upon request	5
	Obesity prevention	2	Training and test splits	4	Not reported	5	Specific methods or models mentioned	6	Not explicitly stated	4
	Obesity management	2	Other cross‐validation split	3	Calibration methods	1	Involved but not detailed	3	Tools mentioned	2
	Obesity treatment	1	Leave‐one‐out cross‐validation	3			General practices or common parameters	2		
			Holdout and validation techniques	2						
			Prototype and expert evaluation	1						
Unsupervised learning (Total: 2)	Another obesity‐related research	1	Not mentioned	1	Not reported	2	Not discussed	1	Explicitly stated	1
	Obesity detection	1	Independent cohort verification	1			Specific methods or models mentioned	1	Not indicated	1

In contrast, areas like obesity prevention, management, and treatment are underexplored, presenting significant opportunities for future research and development. Additionally, there is an inconsistency in the sharing of models and code, which points to a gap in transparency and collaboration within the field. Enhancing openness in sharing resources could improve reproducibility and accelerate advancements. Overall, although AI‐driven obesity detection is well‐established, there is substantial potential for growth in research on prevention, management, and treatment, alongside improvements in methodological transparency and resource sharing.

### 3.1 Narrative Synthesis by Domain

We summarize, by domain (detection, prevention, management, treatment, and other), what studies aimed to do with AI/ML, how they were validated (train/test, *k*‐fold, and external cohorts), and key reporting practices (calibration, hyperparameter tuning, interpretability, and code/model sharing), with brief study‐level examples.

### 3.2 Obesity Detection

Most studies employed supervised learning for risk or status classification, with a minority exploring semisupervised/unsupervised or hybrid approaches. Representative early supervised efforts used basic train/test splits or cross‐validation [11, 12, 13, 14], whereas later work more consistently adopted *k*‐fold validation, explicit holdout strategies, or clearer train/test protocols [15, 16, 17, 18, 19, 20, 21, 22, 23, 24]. Calibration was variably reported—some studies provided performance metrics instead of explicit calibration procedures [15, 20, 21, 25], and a smaller subset explicitly discussed calibration methods [26]. Interpretability began to appear in recent work [23]. Code or model transparency remained limited overall, although several studies noted that it was available upon request or identified the tools used [15, 16, 19, 20]. Notably, a small number moved beyond purely supervised paradigms—e.g., semisupervised or hybrid designs [26, 27, 28]—and one study provided stronger generalizability checks via independent cohort verification [29]. Taken together, detection studies demonstrate a clear trajectory toward more robust validation and reporting; however, calibration and reproducibility practices are still inconsistently documented.

### 3.3 Obesity Prevention

Studies in this domain typically sought to personalize prevention strategies and assess the feasibility of intervention delivery. Prototype‐oriented and expert‐evaluated systems are common [30, 31], with several studies introducing *k*‐fold validation and reporting performance metrics to quantify potential impact [32]. Reporting on calibration is often absent [33, 34], although one contribution explicitly emphasizes the open‐source dissemination of calibration to facilitate reuse and scrutiny [34]. Conceptually, these prevention studies align with individualized, data‐driven approaches (e.g., tailoring recommendations based on user inputs or sensor data); however, methodologically, they would benefit from more consistent validation protocols and clearer calibration reporting.

### 3.4 Obesity Management

Work here focuses on sustained weight control, adherence, and behavior change. Although many studies do not report detailed validation or calibration procedures [5, 35, 36, 37, 38], several signal methodological strengthening via *k*‐fold cross‐validation and explicit calibration methods [39] or alternative cross‐validation splits with model availability upon request [40], and in some cases model availability even without transparent validation reporting [41]. Collectively, management studies suggest a role for AI in adaptive feedback and monitoring; however, the literature would be strengthened by more consistent calibration, transparent hyperparameter tuning, and the routine sharing of code/models.

### 3.5 Obesity Treatment

Evidence is limited, but it shows early progress toward developing treatment guidance and response prediction. A 2023 study [42] illustrates this direction; however, the testing procedures are not reported, and calibration is not documented, echoing the reporting gaps seen elsewhere. Growth in this area will depend on studies that predefine validation plans, report calibration, and improve transparency.

### 3.6 Other Obesity‐Related Research

Adjacent studies explore complementary goals such as biomarker identification and behavioral analytics, using a range of supervised, unsupervised, and hybrid approaches [43, 44]. Some reported performance metrics with structured validation procedures, including other cross‐validation or holdout techniques [7, 43]. Others relied on leave‐one‐out or *k*‐fold validation, although they did not always explicitly report metrics [45, 46]. Notably, several contributions enhanced transparency by providing repositories or making models available [44, 47, 48]. These practices, particularly the use of public repositories, strengthen reproducibility and should be encouraged across all domains.

### 3.7 Implications Across Domains

Consistent with the reviewer's request, our revised results now synthesize what AI/ML is accomplishing within each domain. Detection studies are increasingly focusing on risk stratification and showing a gradual shift toward stronger validation. Prevention studies emphasize prototyping personalized interventions, although calibration and external validation remain limited. Management studies highlight the potential of AI to support adherence and monitor weight trajectories but still lack consistent reporting. Treatment studies are only beginning to emerge and would benefit from more rigorous evaluation frameworks. Finally, other studies illustrate that transparency practices, such as sharing repositories, are both feasible and valuable. Collectively, these patterns have practical implications: AI can tailor preventive strategies, adapt recommendations from wearable and behavioral data, and support sustained engagement through coaching interfaces—provided that validation, calibration, interpretability, and reproducibility are strengthened.

## 4 Risk of Bias Assessment

We also expanded our evaluation to include a formal, domain‐based risk of bias (RoB) assessment aligned with best practices.

Guided by our description of domains used in the data extraction form (Table 3)—participants (D1), predictors (D2), outcome (D3), and analysis (D4)—we operationalized clear signaling questions and decision rules for each domain, ensuring consistency across studies and transparency in judgments.

**TABLE 3:** Description of domains used in the data extraction form.

Domains	Description of domains used in data extraction form	
D1: Participant domain	Covers potential biases related to the selection of participants and data sources used	1.1. Were appropriate data sources used, for example, cohort, randomized controlled trial, or nested case–control study data?
		1.2. Were all inclusions and exclusions of participants appropriate?
D2: Predictor domain	2 Evaluates potential sources of bias from the definition and measurement of the candidate predictors	2.1. Were predictors defined and assessed in a similar way for all participants?
		2.2. Were predictor assessments made without knowledge of outcome data?
		2.3. Were all predictors available at the time the model was intended to be used?
D3: Outcome domain	3 Assesses how and when the outcome was defined and determined	3.1. Was the outcome determined appropriately?
		3.2. Was a prespecified or standard outcome definition used?
		3.3. Were predictors excluded from the outcome definition?
		3.4. Was the outcome defined and determined in a similar way for all participants?
		3.5. Was the outcome determined without knowledge of predictor information?
		3.6. Was the time interval between predictor assessment and outcome determination?
D4: Analysis domain	4 Examines the statistical methods that authors have used to develop and validate the model, including study size, handling of continuous predictors and missing data, selection of predictors, and model performance measures	4.1. Were there a reasonable number of participants with the outcome?
		4.2. Were continuous and categorical predictors handled appropriately?
		4.3. Were all enrolled participants included in the analysis?
		4.4. Were participants with missing data handled appropriately?
		4.5. Was selection of predictors based on univariable analysis avoided?
		4.6. Were complexities in the data (e.g., censoring, competing risks, sampling of control participants) accounted for appropriately?
		4.7. Were relevant model performance measures evaluated appropriately?
		4.8. Were model overfitting and optimism in model performance accounted for?

We then constructed a RoB judgments table (Table 4) at both the domain level (D1–D4) and the overall study level. This table provides the study‐by‐study ratings (low/some concerns/high), enabling replication and facilitating sensitivity analyses.

**TABLE 4:** Risk of bias judgments.

	Risk of bias domains				
Study	D1	D2	D3	D4	Overall
Study 1 [11]	Some concerns	Low	Low	Low	Low
Study 2 [12]	Some concerns	Low	Some concerns	Some concerns	Some concerns
Study 3 [25]	Some concerns	Low	Low	Some concerns	Some concerns
Study 4 [13]	Some concerns	Low	Low	Some concerns	Some concerns
Study 5 [14]	Some concerns	Low	Low	Low	Low
Study 6 [15]	Some concerns	Low	Some concerns	Low	Some concerns
Study 7 [27]	Some concerns	Low	Low	Some concerns	Some concerns
Study 8 [49]	Some concerns	Low	Low	Some concerns	Some concerns
Study 9 [16]	Low	Low	Low	Low	Low
Study 10 [17]	Some concerns	Some concerns	Some concerns	Some concerns	Some concerns
Study 11 [50]	Some concerns	Low	Low	Some concerns	Some concerns
Study 12 [28]	Some concerns	Some concerns	Some concerns	Low	Some concerns
Study 13 [51]	Some concerns	Low	Low	Some concerns	Some concerns
Study 14 [52]	Low	Low	Low	Some concerns	Low
Study 15 [29]	Some concerns	Low	Some concerns	Some concerns	Some concerns
Study 16 [18]	Low	Low	Low	Low	Low
Study 17 [19]	Some concerns	Some concerns	Low	Some concerns	Some concerns
Study 18 [20]	Low	Low	Low	Low	Low
Study 19 [53]	Low	Some concerns	Some concerns	Some concerns	Some concerns
Study 20 [21]	Some concerns	Low	Some concerns	Low	Some concerns
Study 21 [26]	Some concerns	Some concerns	Some concerns	Some concerns	Some concerns
Study 22 [22]	Some concerns	Low	Some concerns	Low	Some concerns
Study 23 [54]	Low	Low	Low	Some concerns	Low
Study 24 [23]	Some concerns	Low	Some concerns	Low	Some concerns
Study 25 [24]	Some concerns	Some concerns	Some concerns	Low	Some concerns
Study 26 [30]	Some concerns	Some concerns	Some concerns	High	Some concerns
Study 27 [31]	Some concerns	Low	Low	Some concerns	Some concerns
Study 28 [32]	Some concerns	Some concerns	Low	Some concerns	Some concerns
Study 29 [33]	Some concerns	Low	Low	Some concerns	Some concerns
Study 30 [34]	High	High	High	High	High
Study 31 [5]	Some concerns	Some concerns	Some concerns	Some concerns	Some concerns
Study 32 [39]	Some concerns	Some concerns	Some concerns	Some concerns	Some concerns
Study 33 [35]	Some concerns	Some concerns	High	High	High
Study 34 [40]	Some concerns	High	Some concerns	Some concerns	Some concerns
Study 35 [36]	Some concerns	High	Some concerns	High	High
Study 36 [37]	Some concerns	Low	Some concerns	High	Some concerns
Study 37 [41]	Some concerns	Some concerns	Some concerns	Some concerns	Some concerns
Study 38 [38]	Some concerns	Some concerns	High	Some concerns	Some concerns
Study 39 [42]	High	High	High	High	High
Study 40 [43]	Low	Low	Some concerns	Low	Low
Study 41 [47]	Some concerns	Low	High	High	High
Study 42 [45]	Some concerns	Low	Low	High	Some concerns
Study 43 [7]	Some concerns	High	High	High	High
Study 44 [46]	Low	Low	Low	Some concerns	Low
Study 45 [48]	Some concerns	Some concerns	High	High	High
Study 46 [44]	High	High	High	High	High

*Note:* D1: participant domain, D2: predictor domain, D3: outcome domain, and D4: analysis domain. Judgments are categorized as low, some concerns, or high.

To improve clarity and align with current reporting standards, we used RobVis, a web application for visualizing RoB assessments in systematic reviews.

**FIGURE 4:**
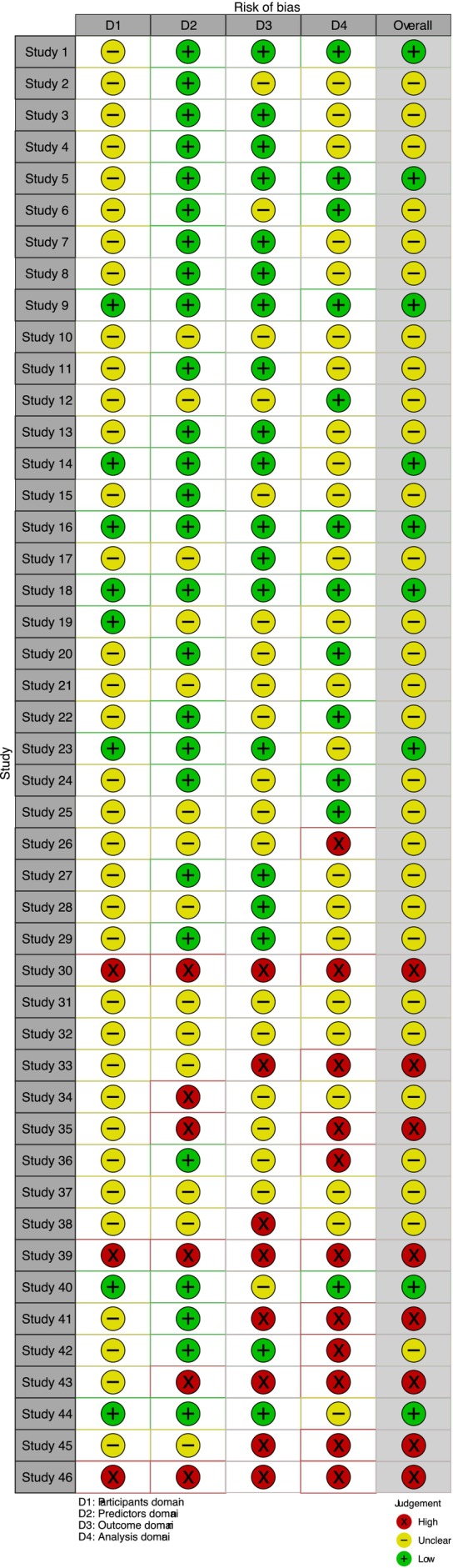
Risk of bias assessment across studies.

**FIGURE 5:**
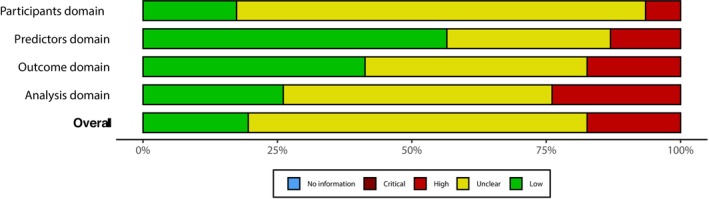
Summary of risk of bias across domains.

The tool generates (i) traffic light plots that display domain‐level judgments for each study (Figure 4) and (ii) weighted bar plots that summarize the distribution of RoB judgments across domains (Figure 5).

The resulting quantitative synthesis is as follows: Across 46 studies, 9 (19.6%) were overall of low risk, 29 (63.0%) had some concerns, and 8 (17.4%) were of high risk. Collectively, the evidence lacks a strong base of low‐risk studies, so any pooled conclusions should be drawn with caution.

As shown in Figure 5, the most frequent weaknesses occur in the participant domain (D1) and the analysis domain (D4). In D1, only eight of 46 studies (17.4%) were low risk. In contrast, 35 (76.1%) had some concerns, and three (6.5%) were high risk, reflecting frequent ambiguity about sampling frames, inclusion and exclusion criteria, and data source representativeness; examples of studies judged as having some concerns include [11, 12, 13, 14, 25, 27, 51]. In contrast, high risk appears in [34, 42, 44].

In D4, the burden of high risk is most significant, with 11 of 46 studies (23.9%) rated high risk, 23 (50.0%) with some concerns. Only 12 (26.1%) were low risk, driven by limited handling of missing data, inadequate control for confounding, and absent or weak internal or external validation; high‐risk examples include studies [30, 34, 35, 36, 37, 42, 47].

By contrast, the predictor domain (D2) is comparatively stronger, and the outcome domain (D3) shows a mixed profile, roughly balanced between low risk and some concerns with fewer high‐risk judgments.

## 5 Discussion

This systematic review of 46 studies published between 2008 and 2024 highlights the growing interest in AI applications for addressing obesity, with a predominant focus on detection rather than prevention or management. The studies employed diverse methodologies, with ML and DL algorithms such as SVMs, random forest, and logistic regression being the most commonly used. Although these technologies demonstrate impressive accuracy and sensitivity in identifying obesity‐related patterns, their application in broader prevention strategies remains limited. A key finding of this review is the emergence of a well‐being‐centric paradigm in obesity research, shifting the focus from isolated detection to holistic health improvement. This approach integrates factors such as mental health, lifestyle habits, and environmental influences, reflecting a more comprehensive understanding of the multifaceted nature of obesity.

Our domain‐organized synthesis reveals a maturity gradient: detection studies dominate and increasingly employ structured validation, whereas prevention/management/treatment remains at early stages with sparse calibration and external validation. These gaps limit translational readiness; preregistered analysis plans, routine calibration reporting, external cohort testing, and open code/model release are priority steps.

However, several barriers hinder the effective implementation of AI in obesity prevention. Data biases, lack of diverse and representative samples, and challenges in model interpretability are significant concerns. For instance, most models rely on retrospective data, which may not capture real‐time dynamics or account for diverse populations. Additionally, the review sheds light on the transformative role of AI in converting Big Data into AI‐ready datasets for nutrition and obesity research. This process involves significant preprocessing, which often requires human judgment and specialized software. The variability in data quality and the lack of standardization across studies remain considerable challenges, emphasizing the need for improved methods and tools to ensure more accurate and reliable results.

One of the key insights from this review is the predominance of AI applications in obesity detection rather than focusing on prevention. Although early detection is crucial for intervention, AI's transformative potential lies in its ability to bridge the gap between identification and proactive prevention. Models can analyze a wide range of risk factors, including dietary patterns, physical activity, and socioeconomic factors, to predict obesity risk and provide recommendations before obesity develops. By harnessing AI's capabilities, healthcare providers can develop targeted interventions that address the underlying causes of obesity, thereby enhancing the effectiveness of prevention strategies.

Another critical aspect discussed is the importance of model interpretability, particularly in healthcare settings, where understanding the rationale behind AI‐driven decisions is crucial for building trust and facilitating more informed decision‐making. AI models are typically powered as “black boxes,” making it difficult for healthcare providers to understand how predictions are made. Although some studies have incorporated explainable AI (XAI) techniques, there is still room for improvement in making these models more accessible to nonexperts. This is crucial, as a deeper understanding of AI outcomes can help healthcare professionals and the public embrace these technologies with greater confidence and widespread adoption. Furthermore, participatory modeling approaches, which integrate expert knowledge with AI techniques, have shown promise in helping to identify and understand the multifaceted factors influencing obesity and well‐being. Many models operate independently and are not specifically designed to interact with healthcare systems. It is crucial to consider how findings from AI research can be effectively translated into practical applications within healthcare systems, ensuring that clinicians are equipped with the necessary tools and knowledge to utilize AI in their practice. Improving compatibility between AI and existing clinical settings is essential to facilitate more adoption.

As AI is increasingly involved in healthcare, it is critical to address ethical concerns, particularly regarding data bias, patient privacy, and model transparency. Many AI models are trained on datasets that may not be fully representative of all populations, leading to biased predictions and disparities in interventions and recommendations. And AI models rely on sensitive personal data, which raises questions about confidentiality and security. Therefore, AI models should be trained on diverse and inclusive data that represent real‐world demographics and backgrounds, and should be accompanied by robust data governance frameworks and regulations to protect patient information. Additionally, AI models should prioritize transparency by making the process accessible and interpretable. Ensuring that AI systems are developed and implemented with a strong emphasis on ethical standards will be vital for fostering public trust and acceptance.

Although AI can act as a decision‐supporting system in reducing long‐term healthcare costs by preventing obesity‐related diseases, initial implementation costs can be substantial and pose significant challenges, such as investment in security and maintenance for data and computing resources. To balance cost‐effectiveness, some options, like optimizing AI algorithms for computing, can reduce development costs. Long‐term cost–benefit analyses can be conducted to evaluate the economic impact of AI in obesity prevention. A thorough understanding of the financial implications associated with implementing AI technologies in obesity prevention can help stakeholders make informed decisions about resource allocation and investment.

The RoB profile (D1–D4) suggests cautious interpretation: most studies had “some concerns,” with recurrent weaknesses in participants (sampling/representativeness) and analysis (missing data, confounding, and validation). Improving transparent cohort construction, principled handling of missingness, prespecified confounders, and layered internal/external validation will raise evidence certainty.

In summary, this review highlights the significant progress made in applying AI to obesity research, particularly in detection, but also reveals considerable challenges that need to be addressed. The potential of AI to transform obesity research and public health is immense, provided that challenges related to data quality, model generalizability, and interpretability are effectively managed. As AI technologies continue to evolve, ongoing collaboration across disciplines will be crucial for unlocking their full potential in improving health outcomes and preventing obesity.

Future research should prioritize longitudinal studies to validate AI‐driven interventions in real‐world settings and examine their long‐term effects on health outcomes. Additionally, expanding datasets to include genetic, metabolic, and lifestyle factors can improve the precision of AI models and enable personalized interventions. Collaborative efforts to share models, datasets, and methodologies will accelerate progress and foster innovation. By addressing these challenges, AI has the potential to revolutionize obesity prevention and contribute to a healthier, more equitable society.

## 6 Conclusion

This systematic review highlights the growing interest in applying AI to address the obesity epidemic, particularly in detection. However, the limited focus on prevention, management, and treatment underscores significant gaps in current research. The integration of AI into obesity prevention strategies requires comprehensive frameworks, interdisciplinary collaboration, and equitable access to technology. By addressing these challenges, AI can serve as a powerful tool in enhancing public health.

## Funding

The authors received no specific funding for this work.

## Conflicts of Interest

The authors declare no conflicts of interest.

## Data Availability

Data sharing not applicable to this article as no datasets were generated or analysed during the current study.
